# Gastroesophageal Reflux Disease (GERD): Highlighting Diagnosis, Treatment, and Lifestyle Changes

**DOI:** 10.7759/cureus.28563

**Published:** 2022-08-29

**Authors:** Pratyaksh Chhabra, Nishikant Ingole

**Affiliations:** 1 Medicine and Surgery, Jawaharlal Nehru Medical College, Datta Meghe Institute of Medical Sciences, Wardha, IND; 2 Pharmacology, Jawaharlal Nehru Medical College, Datta Meghe Institute of Medical Sciences, Wardha, IND

**Keywords:** lifestyle, treatment, diagnosis, gerd, gastroesophageal reflux disease

## Abstract

Millions of people worldwide are affected by the prevalent clinical issue, gastroesophageal reflux disease (GERD). Both conventional and unusual symptoms can identify patients. Many people with GERD benefit from symptomatic relief and are shielded from consequences by acid suppression medication. Our capacity to recognise and manage disease consequences has improved thanks to developments in diagnostic and therapeutic technologies. One of the biggest typical gastrointestinal problems treated by physicians and primary care doctors is GERD, which is characterised by heartburn and regurgitation symptoms. GERD prevalence has increased, especially in North America and East Asia. Proton pump inhibitors (PPIs) have been the cornerstone of medical treatment for GERD for the past thirty years. However, clinicians and patients are becoming more aware of the adverse effects of the PPI class of medications recently.

Additionally, surgical fundoplication has significantly decreased, while the evolution of non-medical therapeutic methodologies for GERD has increased. In the treatment of GERD, lifestyle changes are crucial. Individual variances can be seen in how GERD symptoms change in response to different diets. The study implies that there may be a connection between reflux occurrence and salty foods, chocolates, fat-rich foods, and aerated beverages, even if there is insufficient data to support this theory. In lifestyle modifications, other factors involved are the head of the bed, patients' lying down position, smoking, fat or obesity, and physical exercise. The number of reviews focusing on various diagnostic techniques and treatment modalities is very less, so this review puts emphasis on these areas. This review also covers GERD and its symptoms, epidemiology, and pathophysiology, but significantly focuses on diagnosis, treatment, and lifestyle modification effects.

## Introduction and background

Gastroesophageal reflux disease (GERD) is a gastrointestinal ailment prevalent all over the globe. At some point, about half of all individuals will report reflux symptoms. The Montreal definition of GERD describes it as a state with irritable symptoms and side effects brought on by the reflux of stomach contents into the esophagus [1]. The typical criteria for diagnosing GERD are the classic symptoms and the outcome of an empiric trial with acid suppression. Due to its role in lowering the quality of life and severe morbidity, GERD is a significant health concern. Significant advancements in the quality of life, such as decreased physical affliction, greater vigour, increased physical and social role, and mental well-being, have been linked to the effective treatment of GERD symptoms [2]. Although drugs used in GERD are not extraordinarily costly, managing GERD-affected individuals has been approximated to be two times more expensive than treating the symptoms of similarly situated people who do not have GERD. The excessive morbidity among GERD patients and the higher expense of treating the consequences of GERD that have been improperly treated are probably to blame for this cost disparity. The diagnosis and prognosis of GERD, even in this century with all the technological developments, remains substandard. Surgical intervention in such cases is the last resort as a treatment option. Consumption of fibre-rich diets often proves to be advantageous for the prevention and management of GERD and might add viable years in the affected individual's lifetime [3,4].

## Review

Epidemiology and pathophysiology

GERD is one of the most widespread gastrointestinal conditions, affecting 20% of adults in Western societies. However, because more people have access to over-the-counter acid-reducing drugs, the actual prevalence of this illness may be higher. Males tend to have GERD at a slightly higher rate than females. In contrast to males who are more expected to have erosive esophagitis, females who arrive with GERD symptoms are more likely to have non-erosive reflux disease [5]. However, compared to females, males have had a higher incidence of Barrett's esophagus with long-term GERD symptoms. Older age, an immoderate body mass index (BMI), smoking, anxiety, stress or depression, and insufficient physical activity at work are risk factors for GERD. Consumption habits, for example, the acidity of food to be consumed, and the portion and the schedule of meals, particularly with regard to sleep, may also cause GERD. Recreational exercise appears to be protective, except when done postprandially [6].

**Figure 1:**
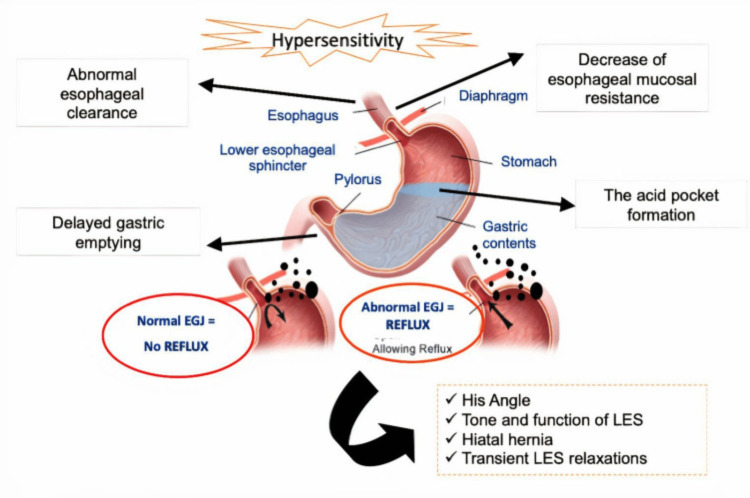
The complex pathogenesis of GERD EGJ = esophagogastric junction; LES = lower esophageal sphincter Image source: Savarino et al., 2021 [7] (Open access)

Lower esophageal sphincter (LES) dysfunction is the leading cause of gastroesophageal reflux; however, other variables may also play a role in its onset. Physiologic and pathologic variables both have a role in the development of GERD. Transient lower esophageal sphincter relaxations (TLESRs) are the most frequent culprit. TLESRs are short-lived, swallow-independent episodes of tone inhibition of the LES. Even though they are physiological, they become more frequent in the postprandial period and are a significant cause of acid reflux in GERD patients. Other concerns include hiatal hernia, decreased esophageal clearance, delayed stomach emptying, and dropped LES pressure (Figure 1) [7]. 

Symptoms

Heartburn is the classic and significant typical symptom of GERD. Reflux of acid into the esophagus causes the feeling of heartburn, a burning sensation in the chest region, which spreads to the oral cavity. Only a few portions of reflux occurrences, nevertheless, show symptoms. Along with regurgitation of the refluxate, a sour taste in the back of the mouth is one more usual symptom of heartburn. Notably, chest discomfort, which is non-cardiac, is often brought on by GERD [8]. Due to the likely dangerous repercussions of chest pain, which is cardiac, it is crucial to determine the principal source of the pain. Heartburn occurring two or more days a week is a symptom that can be used to diagnose GERD. However, symptoms can also be present less frequently. If they are bothersome, they negatively impact well-being. Heartburn frequency and intensity are not related to the extent of esophageal injury. Dysphagia, chest discomfort, water brash, odynophagia, burping, hiccups, nausea, and vomiting are less frequent GERD symptoms [9] Dysphagia is an alert symptom in GERD patients and warrants an upper endoscopy. Patients with chronic heartburn frequently develop dysphagia, which usually worsens over time for meals. Due to their healthy appetites, patients rarely lose weight. However, dysphagia is the initial sign of esophageal cancer but might also show up in Barrett's esophagus. The most typical causes of GERD are peptic stricture and severe inflammation [10,11].

Diagnosis

The clinical history is the primary basis for GERD diagnosis [12,13]. The account must pinpoint the distinctive signs and symptoms, their occurrence, frequency, intensity, aggravating and relieving events, progression through time, and effects on quality of life (Table 1) [14].

**Table 1:** Manifestations of GERD Table source: Henry, 2014 [14] (Open access)

Typical manifestations	Atypical manifestations		
	Pulmonary	Otorhinolaryngological	Oral
Dyspepsia, Regurgitation of acid	Wheezing (chronic), Inflammation of the pharynx, Throat clearing, Pneumonia, Bronchiectasis, Asthma	Sore throat, Otitis, Inflammation of sinuses	Tooth erosion, Halitosis, Aphtha

Diagnostic techniques

Typical, atypical, and extra-esophageal symptoms can be used to categorise the disease's manifestations. Acid regurgitation and heartburn have the highest specificity for GERD [10]. These symptoms can help one make a presumptive diagnosis and start empiric therapy in the absence of alarming symptoms [11]. In some cases, additional diagnostic testing is required to confirm the diagnosis and to look for consequences or other potential explanations of the symptoms. Various diagnostic techniques are discussed below [12,13].

High Digestive Endoscopy

This specific test is the one used most often to evaluate GERD symptoms in people over 40 who have alarming symptoms, for example, difficulty in swallowing, painful swallowing, loss of weight, gastrointestinal bleeding, an urge to vomit (nausea), vomiting, and a history of malignancy in the family. It enables the detection of additional conditions that exhibit symptoms similar to dyspepsias, such as ulcers, especially gastric ulcers, moniliasis of the esophagus, carcinoma of the stomach, and eosinophilic inflammation of the esophagus. It also enables erosions to be observed. It also makes erosions, ulcers, Barrett's esophagus, and peptic stenosis visible. The severity of reflux esophagitis has been categorised in several ways. The Los Angeles classification is the most often used (Table 2) [14].

**Table 2:** Los Angeles endoscopic classification Table source: Henry, 2014 [14] (Open access)

Grade of Reflux Esophagitis	Discovery
A	Single or more erosions minor than 5 mm
B	Single or more erosions bigger than 5 mm in its larger addition, discontinued within esophageal fold apices
C	Contiguous erosions within at least esophageal fold apices, commitment of less than 75% of the esophagus
D	Erosion of minimum 75% of the esophagus circumference

Radiological Esophageal Examination

In diagnosing GERD, this specific examination has limited reactivity and accuracy. It should be requested when the affected individual complains of difficulty swallowing and painful swallowing because it enables a morphological assessment of the esophagus, depicts the presence of stenosis, and identifies a state like a slipping hiatus hernia and an atypical gastroesophageal angle that favours gastroesophageal reflux [14].

Computerised Esophageal Manometry

Esophageal manometry does not serve as a diagnostic tool. Still, it does offer valuable data for assessing esophageal sphincters' pressure tonus and the activity (motor) of the esophageal body part. It can be used to forecast how GERD will develop in the future. The identification and prognosis of critical hypotonia of reduced esophageal detrusor suggest therapeutic continuance therapy, surgery, or fundoplication [14].

Esophageal Scintigraphy

This examination demonstrates GERD caused by technetium-marked contrast consumption. The diagnosis of GERD in children can be made using this non-invasive method. However, this examination is costly and only offered in select high-tech centres [14].

Long-term Esophageal pH Monitoring

This exam determines the diagnosis and severity of GERD in addition to its pattern, such as whether it is prone, positional, or orthostatic. A number of circumstances call for this examination. To diagnose GERD and laryngopharyngeal reflux disease, it is recommended to use a catheter which should at least have two sensorial, one at the far end esophagus and the other at the esophageal detrusor, the upper one or higher. Other recommended procedures are a) GERD prognosis in typical further up endoscopy; b) character of the pattern of gastroesophageal reflux; and c) acid benefaction [15,16]. The identification of reflux disease, which is non-erosive, is found in individuals with standard pH levels and reacts favourably to proton pump inhibitors (PPIs). A patient with regular pH readings, a low symptom score, and an inability to respond to PPI is another common scenario that points to the diagnosis of functional heartburn [14].

Long-Term Wireless Esophageal pH Monitoring (Bravo Capsule)

The benefits of this approach include higher patient comfort, prolonged esophageal pH monitoring (up to 96 hours), and the additional use of preventing catheter shift, which can happen with conventional pH metres. Azzar et al., in 2012, concluded that both the standard and the esophageal pH-metre (wireless) techniques could diagnose pathological gastroesophageal reflux [14].

Bernstein Test

The esophageal mucosa is supplied and imbued with a diluted hydrochloric acid solution in this provocative test. Around 80% sensitivity and specificity are correlated with the onset of symptoms during perfusion. GERD cannot be quantified due to the solely qualitative results. Since the invention of the 24-hours esophageal pH-metre, it has ceased to be utilised [14].

Esophageal Impedanciometry

This novel technique illustrates the refluxate's antegrade and retrograde motions. It assesses the refluxate's physical (gaseous or liquid) and synthetic (non-acidic, acidic, or moderately acidic) characteristics in conjunction with pH-metre. Consequently, this survey characterises the type of refluxate (fluid, flatulent, or varied) as well as whether it is acidic or not. Non-acidic reflux diagnosis in patients who don't respond to PPI is a sign that they need surgery because the fundoplication procedure removes a couple of varieties of reflux [14].

Therapeutic Trials

PPI complete doses may be given for a month together with behavioural treatments to affected individuals below 40 years of age with usual GERD symptoms and no distress or alarm indications (Table 3) [14]. The test is deemed positive when the symptoms go away, and there is a strong indication of GERD [17].

**Table 3:** Behavioural measures for GERD-affected individuals below 40 years of age Table source: Henry, 2014 [14] (Open access)

Sr.No.	Behaviour
1	Bed head elevation
2	Restraint in the consumption of such foods: eatables which are fatty, citric fruits, caffeine, alcoholic beverages and/or aerated drinks, mints, peppermints, tomato, chocolates
3	Particular precaution with high-risk medicines: anticholinergics, theophylline, calcium-channel blockers, alendronate
4	Restraint from reclining in the two hours after eating meals
5	Restraint from bigger meals
6	Quitting smoking
7	Body weight reduction

Treatment

There are a couple of curative ways through which one can proceed towards GERD, clinical and surgical, the preference of which relies on the affected individual's attributes (lifespan, therapeutic adherence, personal inclination, and underlying comorbidity) and components such as medical care reaction, underlying esophageal mucosal erosions, unusual manifestations, and impediments [14,18,19].

Clinical Treatment

Clinical therapy aims to alleviate symptoms, heal esophageal mucosal lesions, and stop problems from occurring. Both pharmaceutical and non-pharmacological measures are the foundation of it [14].

Non-Pharmacological Treatment

Behaviour changes are part of the non-pharmacological treatment (Table 3). Some authors have questioned these recommendations in recent years due to their lack of scientific support and detrimental repercussions on the standard of the patient's life. According to Castro et al., 2000, most GERD patients do not benefit from these therapies [14]. However, these suggestions are well-known and regarded as helpful [20].

Pharmacological Treatment

Different medications can be used to treat GERD. PPIs are the preferred medications because they prevent the stomach parietal cells from producing acid, which lessens the acid's ability to irritate the esophagus. Omeprazole is the most popular. The initial therapy of choice is total PPI dosages for four to eight weeks [14]. A double dose should be administered once before the morning meal and once before the night meal if the patient's symptoms do not disappear [21].

Prokinetic medications and histamine H2 receptor antagonists are regarded as second-line medications. H2 blockers work by inhibiting parietal cells' histamine H2 receptors and lowering acid excretion. Ranitidine, famotidine, cimetidine, and nizatidine are the most widely used. Prokinetic medications speed up stomach unloading but have non-existing results on the momentary loosening of the distal esophageal sphincter. Domperidone and metoclopramide are the most popular. When there is gastroparesis, they must be prescribed [22]. Alginate and sucralfate antacids could be recommended if the outpatient experiences adverse reactions from PPI or histamine H2 sensory receptor antagonists to relieve their symptoms temporarily. Due to these medications' teratogenic effects, pregnant women need to get special treatment. Behavioural interventions must be emphasised, and medicines with systemic absorption should not be used. An antacid regimen is advised [14]. Histamine H2 receptor antagonists may be administered if the symptoms continue. Since most medications are eliminated in milk, only its use among systemic agents is safe during lactation [22,23].

Between 20% to 42% of patients who receive PPI for the treatment of GERD do not respond to it satisfactorily; this condition is referred to as refractory GERD. Moraes Filho states that functional heartburn, non-adherence to therapy, insufficient instructions, variations in the genetic constitution, alkaline gastroesophageal reflux, autoimmune disorders, eosinophilic esophagitis, and wrong diagnosis are the main reasons for refractory GERD [14]. The author speculates that hypersensitivity could be related to GERD, which occurs between the clinical manifestations and exacerbates the signs. Amitriptyline, other tricyclic antidepressants, and serotonin reuptake inhibitors may be prescribed to treat this disease [24,25].

Surgical Treatment

Patients who require ongoing medication, resist protracted treatment, or have complex forms of GERD might consider surgery. Given the potential intervention of PPI in calcium ion uptake, Herbella and Patti argued that interventional therapy should also be designated for adult females going through menopause and those who might be suffering from osteoporosis [14]. Not managing symptoms but maintaining patients' long-term asymptomaticity is the main challenge in clinical treatment [26]. According to Nissen, 1956, the surgical procedure entails creating an anti-esophageal reflux valve utilising the fundus of the stomach (fundoplication) [14]. It corrects an anatomical deficiency by minimising the sliding hiatal hernia seen in 89% of patients suffering from pathological GERD [26]. Furthermore, experimental and clinical studies have shown that it recovers LES competence [27].

**Figure 2:**
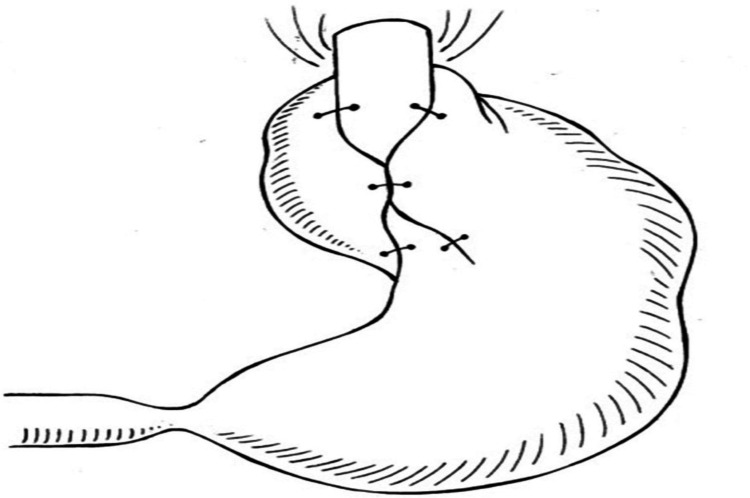
Brandalise and Aranha's fundoplication procedure Image source: Brandalise et al., 1996 [14] (Open access)

Three surgical treatments for GERD are used most frequently, i.e., total fundoplication, in which the esophagus is entirely encircled (360°), partial fundoplication (Toupet), and assorted fundoplication, developed by Brandalise and Aranha (Figure 2) [14]. The decrease in pain experienced after the surgery, quick recovery, early discharge from the hospital, quick integration of everyday undertakings and getting back to jobs, positive aesthetic aspects, and minimum lifestyle modification stand out among the many benefits of video-laparoscopic fundoplication. Additionally, the smaller incision and little postoperative discomfort enable quick diaphragm recovery and early patient deambulation, reducing the risk of respiratory problems [28].

Effects of change in lifestyle in GERD treatment

Studies don't provide enough proof to support the claim that a range of foods can cause reflux. Some evidence shows an association between the development of reflux and salt, foods with excessive salt, chocolates, fat-rich food, and soda drinks. [29,30]. Eating slowly, frequently, and in low quantities should be suggested. Fibrous dietary consumption should be encouraged. Individual variances can be seen in how GERD symptoms change in response to different diets. Therefore, large-scale randomised trials are required to determine whether or not the symptoms of reflux improve when the individual's probable risk factor foods are eliminated from the diet [31].

When in recumbent and side flank posture while lying down, there is an increase in the symptoms of nocturnal reflux. Nocturnal reflux symptoms can be prevented by reclining in the left flank posture and raising the front of the bed when supine. For individuals experiencing reflux manifestations at night, the bed head position must be increased, and the patient must be in the left flank posture [32]. Smoking can be a potential danger for the emergence of GERD manifestations, depending on the amount [33]. The chance of developing GERD is significantly raised by obesity, particularly abdominal obesity and an increase in adipose tissue. Weight loss is advised for GERD patients as fatness is one of the prime risk factors. A crucial risk factor for the expansion of GERD is excessive strenuous exercise. Reflux manifestations are less specific in people who engage in regular, mild-moderate physical activity [34].

Future directions

Due to the perception that PPIs are the only treatment for GERD, drug development in this field has significantly decreased. GERD still has a wide range of unmet needs, which presents a unique opportunity for medication development. Additionally, the increasing number of publications describing various side effects of long-term PPI medication encourages patients to look into alternate therapeutic approaches. Patients may become more interested in antireflux surgical procedures and endoluminal therapy for GERD, which could spur the development of other less invasive non-medical methods. In the treatment of GERD, lifestyle changes are crucial. Individual variances can be seen in how GERD symptoms change in response to different diets. Although there is little proof that lifestyle changes can reduce GERD symptoms [35].

Discussion

GERD is a widespread illness that many patients can successfully manage by amalgamating lifestyle modifications with the right clinical treatment. Most of the patients experience typical symptoms while some of them can also experience atypical symptoms. There are various ways to diagnose GERD, with each methodology having a unique function. It can be hard to manage resistant GERD, which can occur in up to 40% of individuals taking PPIs once a day. The best initial strategy is PPI treatment optimisation. The root causes of PPI failure can be found with in-depth history taking and the application of investigative methods. Drugs like H2 blockers, prokinetics, and baclofen might be utilised in affected individuals with persistent reflux. Neuro-modulators are a crucial component of some therapy strategies for people who face reflux sensitivity or heartburn, which is functional. Even while surgical fundoplication for GERD is still done, it is used much less frequently these days. In a small percentage of patients, endoluminal therapies offer effective symptomatic management and make a good complement to medicinal or surgical care [35,36].

## Conclusions

A serious issue with the digestive system is GERD. With careful consideration of the usual and unusual symptoms, anamnesis is essential for diagnosing GERD (period, intensity, recurrence, aggravating and relieving points, evolution, and effect on the well-being of life). The most accurate diagnostic techniques are high-resolution endoscopy and esophageal peroral endoscopic myotomy (POEM). The therapeutic therapy effectively manages the known symptoms, but the main challenge is maintaining the affected individuals' asymptomatic status over time. Patients who require ongoing medication, are drug-intolerant, or have complex forms of GERD might consider surgery. People with this problem should modify their lifestyle and take medications if required. In the aspect of research, several institutes conducted various clinical trials on many digestive diseases and GERD is one of them.
